# A Randomized, Double-Blind, Placebo Controlled Trial to Determine the Effectiveness a Polyphenolic Extract (*Hibiscus sabdariffa* and *Lippia citriodora*) in the Reduction of Body Fat Mass in Healthy Subjects

**DOI:** 10.3390/foods9010055

**Published:** 2020-01-06

**Authors:** Javier Marhuenda, Silvia Perez, Desirée Victoria-Montesinos, María Salud Abellán, Nuria Caturla, Jonathan Jones, Javier López-Román

**Affiliations:** 1 Faculty of Health Sciences, San Antonio Catholic University of Murcia (UCAM), 30107 Murcia, Spain; sperez2@ucam.edu (S.P.); dvictoria@ucam.edu (D.V.-M.); mabellan@ucam.edu (M.S.A.); jlroman@ucam.edu (J.L.-R.); 2 Monteloeder S.L., 03203 Elche, Alicante, Spain; nuriacaturla@monteloeder.com (N.C.); jonathanjones@monteloeder.com (J.J.)

**Keywords:** obesity, *Hibiscus sabdariffa*, *Lippia citriodora*, polyphenols

## Abstract

The location and quantity of body fat determine the health risks, limiting people with obesity. Recently, polyphenols have attracted the attention of the scientific community because of their potential use for the reduction of obesity. A proprietary formula comprised of a blend of *Lippia citriodora* and *Hibiscus sabdariffa* has been recognized for its high content of polyphenols, powerful antioxidant molecules that may prevent weight gain and could be helpful for the treatment of obesity, as proven previously by in vivo models. The aim of the present study is to determine if the supplementation with *Lippia citriodora* and *Hibiscus sabdariffa* is useful for the treatment of obesity and/or weight control in subjects without a controlled diet. The intake of the extract for 84 days reduced body weight, the body mass index, and the fat mass measured with both bioimpedance and densitometry. This decrease in fat mass was observed to a greater extent, being significant, in the fat mass of the trunk (chest and torso).

## 1. Introduction

The etiopathogenesis of obesity relies on the ability of adipocytes to transform the excess of energy into triglycerides, operating as an energy store. However, the excess of fatty drops in the adipocytes leads to insulin resistance, due to inflammatory responses, which limits their capacity to store the excess of energy [1]. The location and quantity of body fat determine the health risks, limiting people with obesity. That fact has a great impact on several metabolic and chronic diseases, including heart disease, cancer, arthritis, obstructive sleep apnea, hypertension, hyperlipidemia, and type 2 diabetes associated with insulin resistance [2].

This chronic condition of inflammation has especially been related to the generation of insulin resistance. Likewise, the inflammatory response modifies the metabolism of the organism, favoring or suppressing some pathways, such as insulin signaling pathway. That fact, when uncontrolled, may develop into a general inflammatory response than can perpetuate the chronic disfunction of the metabolism observed in obesity [3]. Due to the interest in natural extracts over the last 20 years [4], industry is strongly committed to nutraceuticals, either as a treatment for the disease or as a preventive treatment [5].

Polyphenols are natural extracts that have been extensively studied over the last few years due to their antioxidant and anti-inflammatory capacity, besides their possible role in the prevention and management of several diseases such as cardiovascular diseases, hypertension, diabetes, cancer, or neurodegenerative diseases [6,7,8]. Recently, polyphenols have attracted the attention of the scientific community because of their potential use in the reduction of obesity [9]. The proposed mechanisms include inhibition of the differentiation of adipocytes [10], enhanced fatty acid oxidation [8,11], diminished fatty acid extraction [12], increased thermogenesis and energy expenditure [13], or inhibition of digestive enzymes [14]. However, despite the popularity of this topic, the scientific literature is mainly based on animal and in vitro studies.

The basis of the present work is due to the lack of scientific reports on humans besides the promising use of *Lippia citriodora* and *Hibiscus sabdariffa*, recognized for their high content of polyphenols, powerful antioxidant molecules that may prevent several disease factors such as hypertension, oxidative stress, dyslipidemia, lipid mobilization, or endothelial stiffness [6]. The present *Hibiscus sabdariffa* and *Lippia citriodora* (HS-LC) formula has been previously studied in various clinical studies, in order to help induce weight loss during a controlled diet program [15,16]. The aim of the present study is to determine if the supplementation with *Lippia citriodora* and *Hibiscus sabdariffa* is useful for the treatment of obesity and/or weight control in the absence of a controlled diet.

## 2. Material and Methods

**Figure 1:**
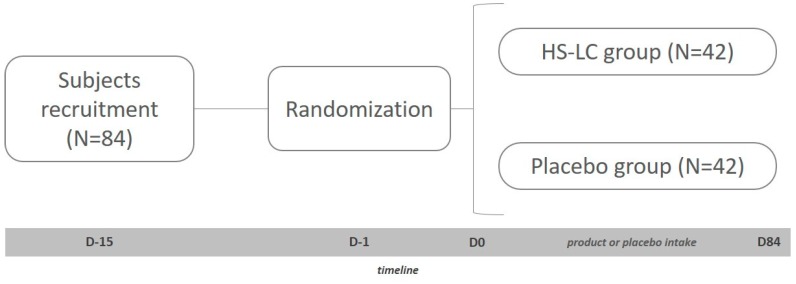
Graphic representation of the study. HS-LC, *Hibiscus sabdariffa* and *Lippia citriodora*

The study consisted of a double-blind, randomized, placebo controlled clinical trial, with two parallel branches of study depending on the extract consumed (experimental or placebo) and single center (Figure 1). A total of 84 sedentary and healthy subjects of both sexes was included in the study after matching all the including criteria (age between 18 and 65 years, both sexes, body mass index (BMI) between 25 and 35 kg/m^2^) and none of the exclusion criteria (illness, pharmacological treatment, toxicological habits, or allergies). After a full disclosure of the implications and restrictions of the protocol, subjects were required to sign the informed consent from. Finally, subjects followed their regular diets during the whole study and were monitored by food consumption diaries.

The extract under study (MetabolAid^®^) consisted of a capsule including a mixture of extracts from *Lippia citriodora* (LC) (325 mg) and *Hibiscus sabdariffa* (HS) (175 mg), both recognized for their abundance in sambubioside derivatives (HS) and verbascoside derivatives (LC) that may help to reduce problems associated with obesity [16]. The placebo capsules contained 500 mg of crystalline microcellulose, maintaining the same aspect as the product under study. MetabolAid^®^ was provided by Monteloeder S.L. (Alicante, Spain) (Patent Application Number P201731147). The polyphenolic composition of the product was quantified and reported in previous studies [17,18].

The present study had a length of 84 days, in which subjects consumed the LC-HS extract or the placebo daily depending on the previous randomization. Therefore, 42 subjects were distributed in the placebo group, and the other 42 subjects were allocated to the LC-HS extract group. Each subject was assigned a code (generated by a number software generator (Epidat v4.1. Epidat, Spain) allocating them to one of the two study groups. Both the researchers and the subjects themselves did not know the composition of the groups.

Subjects came to the research center at the beginning of the study and at the end. At baseline, blood samples were obtained from the cubital veins of subjects from both groups. After blood collection and explaining the operation of the study, subjects received the LC-HS extract or placebo. In order to determine physical activity, every subject was equipped with an accelerometer (ActiGraph wGT3X-BT. ActiGraph, Pensacola, FL, USA) prior to the beginning of the study. Finally, the body composition of the volunteers (total fat mass, fat mass of the trunk, and fat mass of the lower body) was determined by two different methods, bioimpedance (Tanita BC-420M. Tanita Corporation, Arlington Heights, IL, USA) and densitometry (Norland XR-4 using Dual Photonic Absorciometry. Swissray, Edison, NJ, USA).

Moreover, physical activity could partly be a determinant of the possible observed fat loss during the study, due to the importance of exercise with respect to weight control. Therefore, in order to prove if some strategies are effective for the treatment of obesity, physical exercise must be measured and regulated. For this purpose, physical exercise was measured by the metabolic equivalent of task (MET), which led to the determination of metabolic equivalents, relating the intensity of physical activity to the kilocalories consumed by subjects. MET values were determined in accordance with the “Compendium of Physical Activities” [19].

Finally, blood analysis was performed for the monitoring of glycemic (glycemia and glycated hemoglobin) and lipidemic parameters (total cholesterol, low density lipoprotein (LDL), high density lipoprotein (HDL), and total triglycerides). The Reflotron Plus system (Roche Diagnostics S.L., Sant Cugat del Vallès, Barcelona, Spain) was used to obtain the glycemic and lipidemic values.

The study was conducted in accordance with the Declaration of Helsinki (randomized controlled trials registration number: NCT04105192), and the protocol was approved by the Ethics Committee of UCAM (CE011815).

## 3. Statistical Determinations

A descriptive analysis (mean and standard deviation) of all the variables under study was carried out. For quantitative variables, *t*-Student comparisons were developed between both the placebo and LC-HS extract groups. The qualitative variables were analyzed by means of a homogeneity test based on the Chi squared distribution when the expected values made it possible and by Fisher’s exact test otherwise. To analyze the differences between the groups (LC-HS extract and placebo), an analysis of variance for repeated measures with the intrasubject factor (baseline and at the end, after 84 days) and intersubject factor (experimental group and placebo group) was carried out. The Bonferroni test was performed for the post-hoc analysis, using 0.05 as the level of significance. Statistical analysis was carried out with the SPSS 21.0 software (IBM. New York, NY, USA).

## 4. Results and Discussion

### 4.1. Blood Parameters

As shown in Table 1, blood parameters showed similar levels both at baseline and at the end of the study (*p* > 0.05).

There was a downward trend for cholesterol and LDL values after the consumption of the product under study. However, the LC-HS extract did not improve (*p* > 0.05) the lipid profile of the subjects. In fact, the evolution of the lipid profile was similar between subjects consuming the placebo and those who consumed the LC-HS extract (Table 1). As observed for the lipid profile, the glycemic parameters showed similar values along all the study, regardless of the consumption of the placebo or the LC-HS extract (Table 1).

### 4.2. Weight and BMI

In order to determine the relevance of sex in the results obtained in the present research, we developed a sex based study of weight loss. Interestingly, men showed slightly high weight loss after treatment with LC-HS extract than women (90.03 ± 15.76 kg for men and 77.87 ± 77.05 kg for women at baseline compared to 89.02 ± 10.46 kg in the case of men and 77.05 ± 10.44 kg for women at the end of the study), but not for the placebo. However, neither weight nor BMI showed relevant changes, leading to no statistically significant variation (*p* > 0.05).

**Figure 2:**
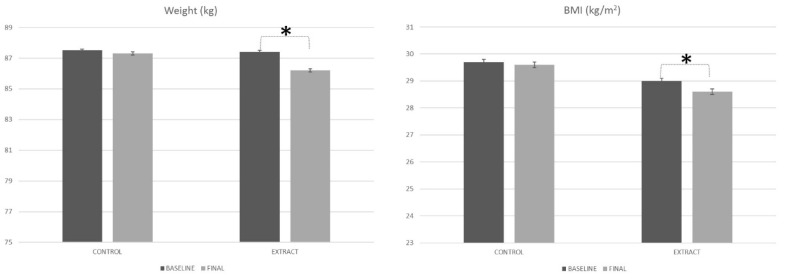
Weight and BMI evolution along the study. * Means statistically significant differences.

The weight of the subjects at the beginning of the study showed similar values for both the placebo and LC-HS extract. Placebo group subjects started the study with an 87.5 ± 15.8 kg body weight, while subjects who were included in the LC-HS extract group showed a total of 87.4 ± 11.1 kg body weight (Figure 2). When comparing the weight of the volunteers of both groups under study, no statistically significant differences were observed (*p* = 0.972); hence, it can be stated that the weight of all the subjects under study was similar at the beginning of the present study.

After 84 days of treatment, the weight of subjects from the placebo group was 87.3 ± 15.3 kg (Figure 2), which represents a similar body weight as that observed at baseline (*p* = 0.479). On the other hand, after 84 days of treatment with the experimental LC-HS extract, the body weight of subjects was 86.2 ± 10.9 kg. That change in the body weight of volunteers consuming the polyphenol rich LC-HS extract for 84 days represented a statistically significant variation (*p* < 0.001) compared to the body weight observed at baseline in these subjects. Therefore, it can be determined that the LC-HS extract significantly decreased the body weight of the subjects (*p* = 0.014).

Similar to what was observed previously for body weight, the BMI of the subjects at the beginning of the study showed similar values (*p* = 0.436) for the placebo group (29.7 ± 4.7 kg/m^2^) and HS-LC group (29.0 ± 3.1 kg/m^2^). After the consumption of both the placebo and the LC-HS extract for 84 days, the BMI was 29.6 ± 4.4 kg/m^2^ and 28.6 ± 3.0 kg/m^2^ for the placebo and LC-HS extract, respectively. Therefore, the diminution of BMI after the consumption of the placebo was shown to be less than that observed for the LC-HS extract (*p* = 0.013).

Despite the lack of in vivo studies found in the scientific literature, the antiobesogenic properties of LC-HS has been previously reported in some in vitro models and animal models. For one study using an insulin resistant hypertrophic 3T3-L1-adipocyte model, the polyphenols from HS were shown to inhibit the increase in triglycerides, oxidative stress due to free radicals, and inflammatory adipokines [18,20]. In addition, the administration of HS in the form of an extract has also been able to prevent hepatic steatosis by regulating the expression of various genes responsible for glycemic and lipid homeostasis [21], as well as reducing blood pressure and improving vascular endothelial function [11] in hyperlipidemic mouse models [22]. Finally, despite HS being a rich polyphenolic matrix, the scientific literature seems to point to quercetin-3-O-β-D-glucuronide and its aglycone as the main ones responsible for the effects described [23].

In the same way, other studies of LC and its content in polyphenols showed promising effects in the reduction of fatty tissue, such as decreased lipogenesis, enhanced oxidation of fatty acids, and the activation of the AMP-activated protein kinase (AMPK) pathway (probably through the activation of the peroxisome proliferator-activated receptor (PPAR-gamma) receptor and adiponectin) [24]. In the same way as the HS extract, the treatment with LC in an animal model with hyperlipidemia prevented fatty liver disease and improved lipid metabolism. In fact, it seems that LC-HS can have synergistic effects for the treatment of fatty liver and lipid metabolism [22,24]. A recent study has revealed the joint ability of these two plant matrices to treat obesity, improving the metabolism of hyperlipidemic rats through the increase of thermogenesis inducing genes in white adipose tissue and correlating with the increase in AMPK phosphorylation and fatty acid oxidation at the liver level [16]. Moreover, in one of the few in vivo studies with humans, it was reported that the combination of LC-HS can modulate the response of incretins, related to the regulation of appetite and hunger, which could be beneficial in the treatment of obesity in the context of an isocaloric healthy diet [15].

Finally, Herranz-López and Barrajón-Catalán [24] studied the effects of LC in an insulin resistant hypertrophic 3T3-L1-adipocyte model and reported a decrease in lipogenesis, improvement of fatty acid oxidation, and activation of the energy regulator pathway regulated by AMPK. They suggested a mechanism activated by transcriptional factors such as reactive oxygen species (ROS) mediated downregulation of nuclear factor kappa-B transcription factor (NF-κB) and peroxisome proliferator activated receptor gamma (PPAR-γ) dependent transcriptional upregulation of adiponectin [25].

### 4.3. Body Composition

#### 4.3.1. Bioimpedance

Weight reduction is necessary for the treatment of obesity. However, some treatments may cause a reduction on the muscular mass, reducing metabolic waste, and compromising weight loss and/or maintenance of the lost weight [26]. In order to guarantee that the loss of body weight is accompanied by the loss of fat mass and the maintenance of muscular mass, we performed two different analyses of body composition.

Bioimpedance is a secure, easy, and non-invasive method that can be used for the measurement of body composition [27]. However, the values must be considered carefully due to the limited reliability of bioimpedance. Intracellular water alterations are frequent in protein-calorie malnutrition, and therefore, the non-fatty mass measures do not exactly reflect the amount of real body composition [27]. Ellis and Bell [28] described a series of general recommendations for the use of impedance after its implementation became widespread by a large number of researchers and has not always been well used. In general, the impedance of legs and arms is less predictive of non-fatty mass than full-body bioimpedance [29].

**Figure 3:**
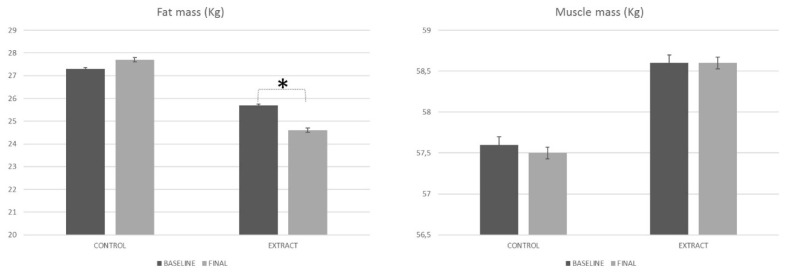
Body composition evolution along the study, measured by bioimpedance. * Means statistically significant differences.

After the 84 days of treatment, the values obtained in the present study (Figure 3) showed a reduction in the fat mass in the case of the LC-HS extract group (*p* < 0.001), but no differences were observed for the placebo group (*p* = 0.309). Differences between both groups were also statistically significant (*p* < 0.002), exhibiting a remarkable capacity of the LC-HS extract to reduce body fat mass. Moreover, it was complemented by the maintenance of muscle mass, as observed in the Figure 3.

#### 4.3.2. Densitometry

Due to the limited reliability of bioimpedance, the analysis of body composition was repeated using densitometry equipment. Dual-energy X-ray absorptiometry (DXA) has been generally used for clinical purposes to measure bone mineral content and bone mineral density as part of osteoporosis evaluation [30]. More recently, densitometry has gained popularity for other purposes due to its ability to measure body composition, incorporating measures of whole body and regional lean mass and fat mass, including visceral adipose tissue [31,32].

**Figure 4:**
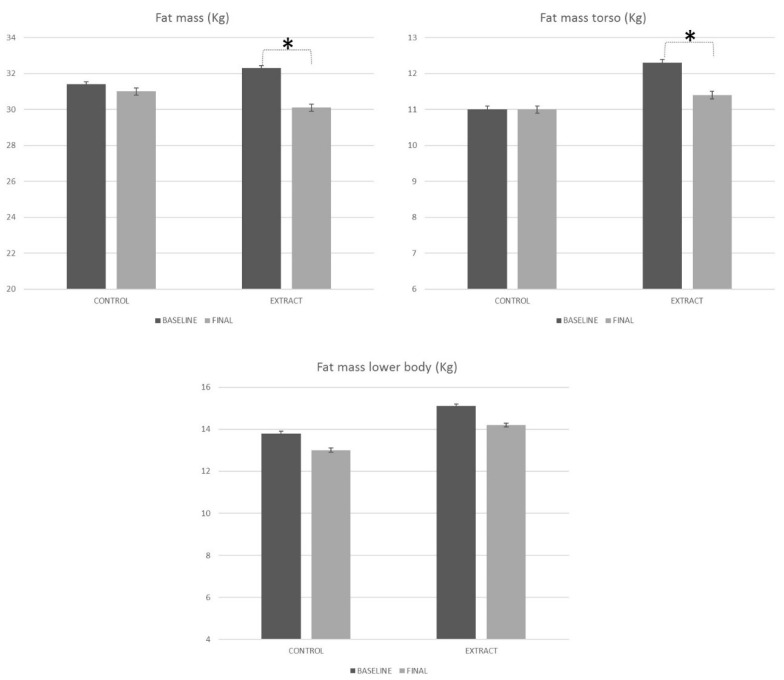
Body composition evolution along the study, measured by densitometry. * Means statistically significant differences.

The total fat mass of the subjects at the beginning of the study showed similar values for both the placebo and LC-HS extract groups. The placebo group subjects started the study with a 31.4 ± 1.8 kg fat mass, while subjects who were included in the extract group showed a total of 32.3 ± 1.7 kg fat mass (Figure 4). When comparing the fat mass of the volunteers of both groups under study, no statistically significant differences were observed (*p* = 0.713), so it can be stated that the fat mass of all the subjects under study was similar at the beginning of the present study.

At the end of the study, the fat mass of subjects from the placebo group was 31.0 ± 1.7 kg (Figure 4), which represents a similar fat mass as that observed at baseline (*p* = 0.361). In turn, the fat mass of subjects consuming the LC-HS extract was statistically less (*p* > 0.001) compared to that observed at baseline (30.1 ± 1.7 kg), which represents a 6.9% decrease with respect to baseline. Interestingly, the differences observed in the reduction of fat mass showed the effectiveness of the HS-LC extract in reducing fat mass.

Fat deposits observed in the obese population determine comorbidities and many related illnesses [33]. Central fat deposits have been particularly associated with alterations in various metabolic pathways more than peripheral fat, being most evident when intra-abdominal visceral fat deposits increase [34]. Visceral obesity has also been associated with endocrine abnormalities, especially with regard to cortisol, growth hormones, and sex steroids, with a profound impact on the activity of these hormones in peripheral or white tissues [33]. Individuals with visceral obesity and/or metabolic syndrome virtually present all hormonal alterations that occur in old age, suggesting that this condition determines a kind of premature aging [34].

In the present study, the placebo group did not reduce fat mass neither in the torso (*p* = 0.913) nor the lower body (*p* = 0.391). Regarding the intake of the LC-HS extract, the results from the densitometry analysis of the body weight showed a statistically significant diminution of the fat mass allocated in the torso (*p* = 0.001) and a minor reduction in the lower body (*p* = 0.306) (Figure 4).

Judging by the evolution of both groups and the difference observed (*p* = 0.018), the LC-HS extract was more effective at decreasing fat mass in the torso after 84 days of treatment, compared with placebo. However, despite reducing fat mass in the lower body, the results were not statistically significant for the placebo or the LC-HS extract. As observed, treatment with the LC-HS extract for 84 days, but not placebo, led to the reduction of central fat mass, which could reduce the comorbidities associated with obesity and the related inflammation.

Of all the proposed mechanisms, the regulation of AMPK expression appeared to be the main molecular target of the action of LC-HS, allowing glycidic and lipid regulation in the body [16,24]. Activation of AMPK increases the biogenesis and density of mitochondria, which would lead to an increase in the maximum and basal metabolic rate, as well as tolerance to physical activity [35]. In fact, the increase of AMPK expression in mice seems to be decisive to increase voluntary physical activity [36]. Finally, AMPK also improves the metabolic rate by activating phosphofructokinase-2, increasing gluconeogenesis, and reducing the sensation of appetite [37].

Therefore, the activation of AMPK by HS and LC could increase the basal metabolic rate, increasing energy expenditure. This would lead to an improvement in body composition, necessary for the treatment of obesity [38]. Finally, the activation of AMPK in certain regions of the hypothalamus responsible for the control of food intake decreases the feeling of appetite and increases the action of leptin [39].

### 4.4. Physical Activity

**Figure 5:**
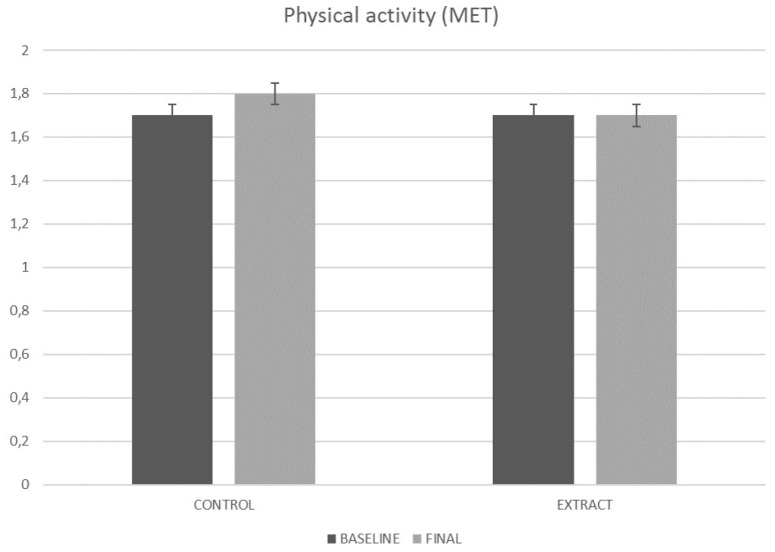
Physical activity EVOLUTION along the study. MET, metabolic equivalent of task.

In order to reduce the error caused by personal differences in physical activity [40], sedentary volunteers were exclusively recruited. The measurement of METs by the accelerometer revealed that subjects from both placebo and experimental groups maintained the same physical activity during the 84 days of the study (Figure 5). Regarding placebo group, values ranged (*p* = 0.418) from 1.7 ± 0.3 MET at baseline to 1.8 ± 0.3 MET at the end of the study. In turn, the experimental group showed similar values (*p* = 0.842) both at baseline 1.7 ± 0.4 MET and at the end of the study 1.7 ± 0.3 MET. That fact supports that changes observed in subjects consuming the LC-HS extract may be a consequence of such consumption and not a change in physical activity habits. 

## 5. Conclusions

The daily consumption of the LC-HS extract, but not the placebo, for 84 days was able to reduce body weight, BMI, and central fat mass, regardless of the physical activity. The LC-HS extract seems to affect only weight variables due to possible changes in molecular pathways, but its impact on biochemistry blood parameters such as cholesterol, LDL, HDL, and triglycerides needs more study.

## Figures and Tables

**Table 1:** Evolution of biochemical blood parameters of subjects during the study.

		Baseline	Final
Total Cholesterol	Control	228.2 ± 6.2	232.8 ± 9.5
	EXTRACT	234.9 ± 6	223.2 ± 9
HDL	Control	56.5 ± 2.5	58.9 ± 2.4
	Extract	58.5 ± 2.1	60.6 ± 2.2
LDL	Control	144.1 ± 6.3	144.17.6
	Extract	134.1 ± 7.2	133.8 ± 7.5
Triglycerides	Control	138.8 ± 12.1	134.1 ± 612.4
	Extract	136 ± 612.4	133.1 ± 12.4

Values are expressed in mg/dL.
