# Correction: Marhuenda, J., et al. A Randomized, Double-Blind, Placebo Controlled Trial to Determine the Effectiveness a Polyphenolic Extract (*Hibiscus sabdariffa* and *Lippia citriodora*) in the Reduction of Body Fat Mass in Healthy Subjects. *Foods* 2020, *9*(1), 55

**DOI:** 10.3390/foods9030279

**Published:** 2020-03-03

**Authors:** Javier Marhuenda, Silvia Perez-Piñero, Desirée Victoria-Montesinos, María Salud Abellán-Ruiz, Nuria Caturla, Jonathan Jones, Javier López-Román

**Affiliations:** 1 Faculty of Health Sciences, San Antonio Catholic University of Murcia (UCAM), 30,107 Murcia, Spain; sperez2@ucam.edu (S.P.-P.); dvictoria@ucam.edu (D.V.-M.); mabellan@ucam.edu (M.S.A.-R.); jlroman@ucam.edu (J.L.-R.); 2 Monteloeder S.L., 03203 Elche, Alicante, Spain; nuriacaturla@monteloeder.com (N.C.); jonathanjones@monteloeder.com (J.J.)

The authors wish to make the following correction to this paper [1]:

There are text errors in Section 4.3.2 Densitometry, third paragraph. The second sentence states that “...LC-HS extract was statistically less (*p* > 0.001) compared...”. It should instead read “...LC-HS extract was statistically less (*p* < 0.001) compared... ”. Furthermore, the last sentence of the same paragraph reads “Interestingly, the differences observed in the reduction of fat mass showed the effectiveness of the HS-LC extract in reducing fat mass”. It should instead read “Interestingly, the differences observed in the reduction of fat mass showed the effectiveness of the LC-HS extract in reducing fat mass”. In this respect, there are several parts of the manuscript which reads HS-LC, it should be read as LC-HS instead. This includes: page 2, paragraph 2; Figure 1; page 4, last paragraph.

There is also a missing explanation in the same section, regarding the intergroup analysis of the results. At the end of the third paragraph, it should include the following sentence: “Intergroup analysis revealed significant differences between both groups, where LC-HS significantly decreased body fat mass compared to placebo (*p* < 0.003)”.

In Section 4.2 Weight and BMI, the sentence “Therefore, it can be determined that the LC-HS extract significantly decreased the body weight of the subjects (*p* = 0.014)”, should read “Inter-group analysis revealed that the LC-HS extract significantly decreased the body weight compared to placebo (*p* = 0.014)”. In the same section, sentence “Therefore, the diminution of BMI after the consumption of the placebo was shown to be less than that observed for the LC-HS extract (*p* = 0.013)”, should read “Therefore, intergroup analysis revealed that the BMI of the LC-HS group significantly decreased with respect to the placebo group (*p* = 0.013)”.

Information is missing from Figure 2. The original figure is as follows: 

**Figure 2:**
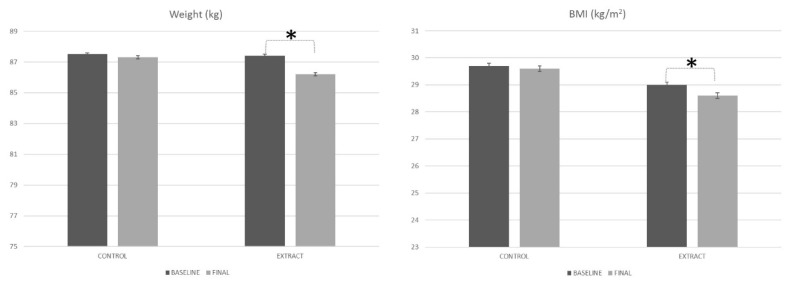
Weight and BMI evolution along the study. * Means statistically significant differences.

It should be as follows:

**Figure 2:**
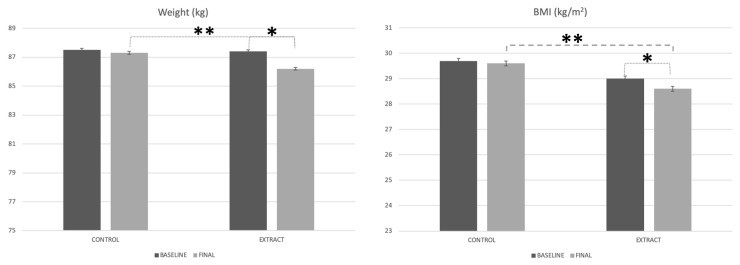
Weight and BMI evolution along the study. * Mean statistically significant differences. ** Mean statistically significant differences between groups.

Information is missing on Figure 4. The original figure is as follows:

**Figure 4:**
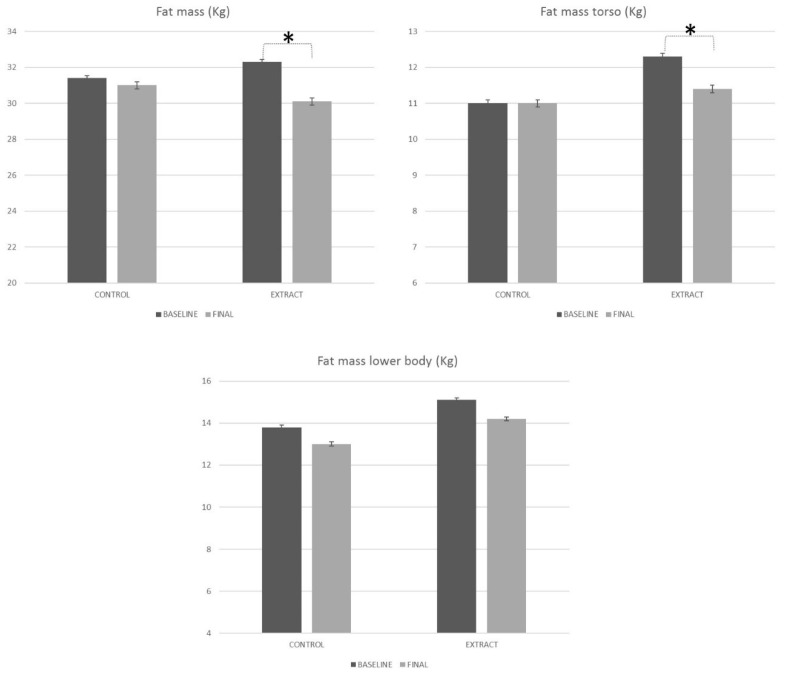
Body composition evolution along the study, measured by densitometry. * Means statistically significant differences.

It should be as follows instead:

**Figure 4:**
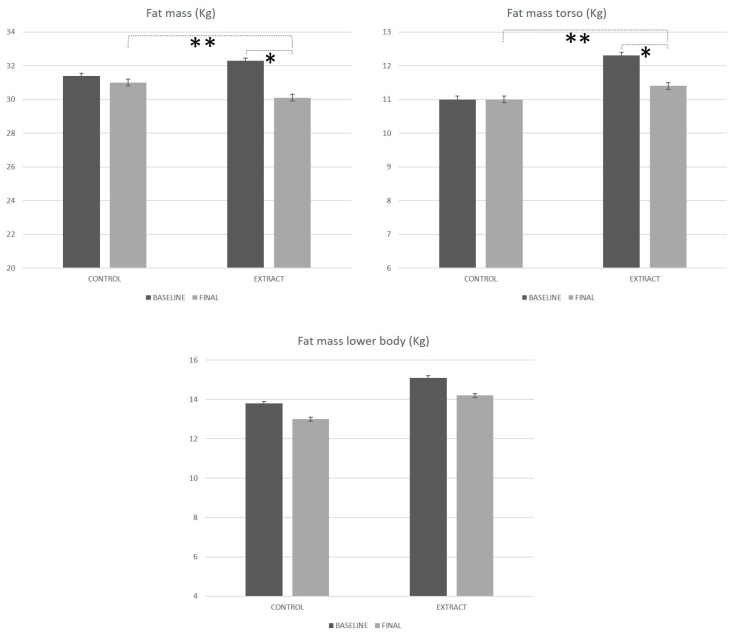
Body composition evolution along the study, measured by densitometry. * Means statistically significant differences. ** Mean statistically significant differences between groups.

These changes have no material impact on the conclusions of our paper. 

The authors would like to apologize for any inconvenience caused to the readers by these changes.
